# The Role of Microbes in Ensiling

**DOI:** 10.3390/microorganisms13102237

**Published:** 2025-09-24

**Authors:** Olli H. Tuovinen, Seppo I. Niemelä, Päivi J. Rajala-Schultz

**Affiliations:** 1 Department of Microbiology, Ohio State University, 484 W. 12th Avenue, Columbus, OH 43210, USA; 2 Department of Applied Chemistry and Microbiology, Division of Microbiology, Biocenter Viikki, Helsinki University, 00014 Helsinki, Finland; sepponiemela89@gmail.com; 3 Department of Production Animal Medicine, Faculty of Veterinary Medicine, Helsinki University, Paroninkuja 20, 04920 Saarentaus, Finland; paivi.rajala-schultz@helsinki.fi

**Keywords:** ensiling, lactic acid bacteria, mycotoxins, pathogens, silage

## Abstract

Ensiling is the microbial processing of forage, based on the fermentation of plant sap that acidifies the silage to pH < 5. This acidity threshold prevents microbial activity that could otherwise produce inhibitory, toxic, malodorous or otherwise undesired metabolites. Anaerobic conditions are key to silage production and storage in silos because aerobic exposure would change the microbiota to counterproductive metabolism. This review outlines the principal microbial groups involved in the open ensiling process and discusses some additive effects with externally added compounds that have been used in this industry to enhance anaerobiosis, lactic acid fermentation, preservation, and safe storage. The ensiling process and the type of forage in the silage may impact methanogenesis in ruminants, and additional effects on ruminal digestion have also been reported.

## 1. Introduction

**Figure 1:**
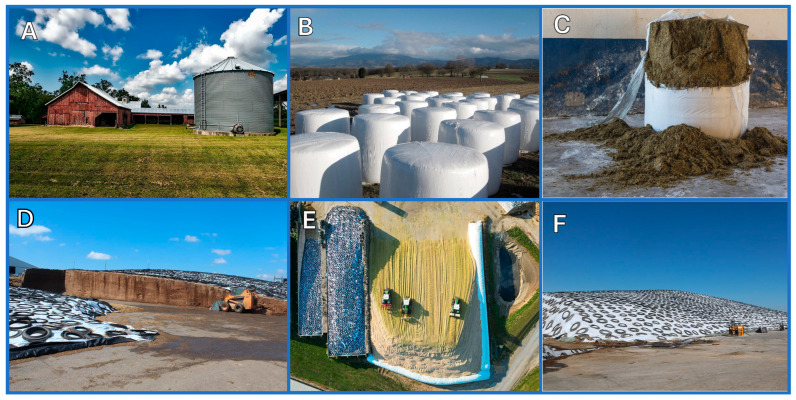
Main silo types: (**A**) tower silo; (**B**,**C**) silage bags; (**D**–**F**) bunker silos, with recycled tires as weights on the plastic sheets. (Photos **A**–**C** courtesy via Pixabay, photos **D**–**F** courtesy of the American Dairy Association Mideast).

Silage is a term used to describe green or wet crops preserved by anaerobic fermentation for livestock feed. Usually, silage is based on mixed species of grass (e.g., ryegrass, timothy, fescues) and legumes (e.g., clover, lucerne). Cereal crops, most notably corn, are also common in silage making but they have a relatively low protein content as compared to leguminous silage [1]. Specific crops, such as corn and sorghum, are sometimes used as sole feed material. Cultivar and harvest time are also taken into consideration when determining the silage treatment. Haylage is a silage product that is made specifically from forage grasses and legumes and contains 40 to 60% moisture. The process of microbial fermentation of herbage to organic acids is termed ensilage, and the fermentation takes place in silos, which have different designs: tower silos, bunker silos, silage bags, and silage piles (Figure 1). They vary in capital investment, technical aspects, construction material, constraints, and specific choice factors such as the cost, capacity, herd size, mechanization, storage loss of dry matter, and safety margins. Extension services in many U.S. land-grant universities have websites for silo designs, ensiling, and silage (e.g., [2,3,4,5]).

The purpose of this paper is to provide a concise, narrative review of microorganisms associated with silage and their roles in stabilizing its nutritive value for animals. Dairy and cattle as food animals constitute a large part of human nutrition, and sustainable silage is, therefore, an important aspect of livestock husbandry. The relevant literature was searched on the internet and Google Scholar, checking references cited in recent papers and browsing publications in special silage topic collections in scientific journals, symposium proceedings, and chapter books. Multiple search words and their combinations were used to extract relevant information.

Anaerobic conditions are crucial for stabilizing the nutritive value of silage. Aerobic respiration leads to microbial decomposition of plant biomass, resulting in the biodegradation of plant polymers and formation of gases (mostly carbon dioxide) and volatile metabolites, all counterproductive to preserving dry matter and nutrients in silage [6]. It may also lead to products that are toxic in the digestive system. In the initial phases of ensilage, oxygen trapped in freshly harvested herbage is rapidly depleted by respiratory enzymes of plant tissues.

## 2. History

The reported roots of ensiling and processed silage targeted for animal feed date back to the 1800s in Europe [7], but little information is available to assess the success of ensiling. Ensiling silage was briefed in 1877 in a French booklet by Auguste Goffart, followed by its English translation in 1879 [8,9]. Goffart’s description referred to alcoholic and acetate fermentations and putrid spoilage due to butyrate fermentation, but no specific microorganisms were mentioned. After 30 years, Crisp and Patterson [10] summarized silage-making practices and technical specifications in Maryland. In the early 1900s and during the years between World War I (WWI) and World War II (WWII), lactic acid bacteria were tested for silage in infrequently documented instances mostly in Europe [11,12,13,14], but random information including the sources of bacteria is fragmentary and obscure and the biology of lactic acid bacteria was inadequately understood. The role of temperature in ensiling was recognized as very important, and two common regimens were noted, namely warm fermentation (approx. 50 °C) and cold fermentation (max. 30 °C) [11], each with specific forage filling, compaction, advantages, and disadvantages. Following WWII, increasing demand for more intensive animal production and cost-effectiveness of cattle feed energized interest in improving the silage process and mechanization [7,15,16,17]. Molasses and other readily digestible supplements were tested in efforts to promote bacterial fermentation in silage. Inoculation experiments with lactic acid bacteria started to address parameters such as the extent and rate of silage inoculation, viability and activity, source inoculum and plant variety, natural bacterial community, and temperature. Inoculation experiments including farm-scale trials and pilot- and commercial-scale silos confirmed positive effects of lactic acid bacteria on silage quality [14,15,18]. These developments had already led, in the 1970s, to commercially available bacterial starter cultures in the agricultural market.

## 3. Fermentation

The ensiling process has distinct phases based on the dominant microbial activity (Table 1). Enterobacteriaceae are often involved in the initial stages of ensilage because of their association with aerial parts of forage following field application of manure and liquid manure. They are facultative anaerobes, usually non-pathogenic, and can utilize a wide range of carbohydrates for fermentative metabolism [19,20]. Enterobacteriaceae detected in forage include *Klebsiella* spp., *Escherichia coli*, *Citrobacter* spp., and *Erwinia herbicola*. Enteric bacteria in anaerobic zones in silage are fermentative and active in ammonification (ammonia release from amide and amine groups) from anaerobic breakdown of plant material [21,22,23]. Some of these metabolites are undesirable (e.g., products of mixed acid fermentation, ammonia from protein decomposition) as they inhibit or divert the flow of carbon away from lactic acid bacteria [24]. The domination of Enterobacteriaceae is usually less than a week, as streptococci and leuconostocs gradually replace them. When the pH drops below 5 during lactic acid fermentation, there is a shift in the population toward the predominance of lactobacilli and pediococci, and the homofermentative pathway (Table 1) becomes dominant.

Lactic acid fermentation (Table 2) is of major importance in silage production [21,22,25,26]. High quality silage contains more lactic acid than any other acid, up to 70% of total acids. Lactic acid concentrations approaching 70 g/kg dry matter have been reported for some silage with additives, but ten-fold less variation has also been noted [27]. Acetic, propionic, and succinic acids are also desired fermentation products. Acetic acid is, however, also formed under aerobic conditions [28]. Mixed acid fermentation yields multiple products depending on the bacterium. It is undesired because it competes with the lactic acid pathway for the substrate. Under aerobic conditions, acetic acid is formed by the oxidation of glucose and ethanol, but acetic acid bacteria also oxidize many other alcohols and carbohydrates, again undesired because of the loss of carbon as CO_2_ and competition with lactic acid pathway. Lactic acid producing bacteria, assigned in the genera *Lactobacillus*, *Streptococcus*, *Pediococcus*, *Leuconostoc*, and *Enterococcus* are facultative anaerobes commonly found in forage crops [21,29]. Because lactic acid bacteria are not always found in large numbers in some herbage, they are sometimes added as silage inoculants to promote lactic acid fermentation and to suppress enteric bacteria, yeasts, and filamentous fungi [30].

**Figure 2:**
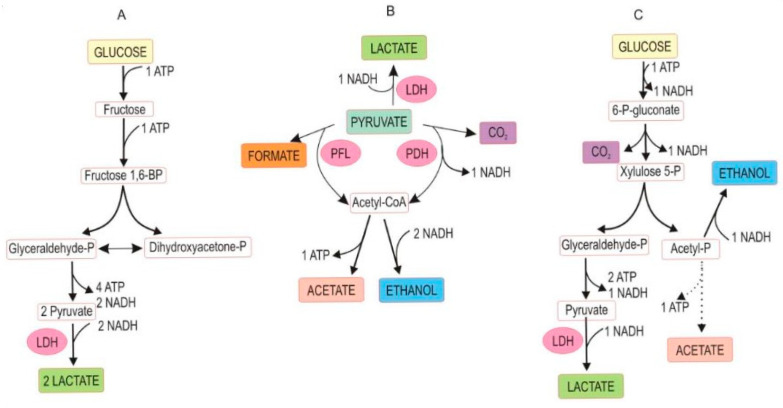
Abridged pathway of glucose fermentation by lactic acid-producing bacteria. (**A**) Homofermentation; (**B**) mixed acid fermentation; (**C**) heterofermentation. Abbreviations: P = phosphate, BP = bisphosphate, PDH = pyruvate dehydrogenase, PFL = pyruvate formate lyase, LDH = lactate dehydrogenase. (With permission, A.O. Ojo and O. de Smidt [31], ©2023). Mixed acid fermentation can also produce succinic acid. Note the loss of carbon as CO_2_ in (**B**,**C**) from the carbon balance. All pathways are coupled with the formation of reducing power (NADH) and metabolic energy (ATP).

The fermentation occurs under anaerobic conditions and is based on naturally available sugars (mostly glucose and fructose) in plant sap. Plant fiber biodegradation, yet undesired because it causes a loss of dry matter, also contributes to soluble sugars. Glycolysis splits glucose into two equivalents of pyruvic acid, which then leads to three major metabolic pathways that can prevail in silage: homofermentation, heterofermentation, and mixed acid fermentation, schematically outlined in Figure 2 [31]. Other water-soluble carbohydrates in plant sap serving as fermentative substrates are disaccharides (e.g., sucrose, melibiose), tri- and tetrasaccharides (e.g., raffinose, stachyose), and polysaccharides (e.g., fructans) [32]. Silage may be lacerated, minced, or chopped herbage to liberate plant sap for rapid lactic acid fermentation. The microbiological processes and fermentative reactions in ensilage are like those occurring in sauerkraut fermentation and dill pickle.

## 4. *Lactobacillus* Starter Cultures

Many approaches with inoculants have been tested over the decades for promoting lactic acid fermentation and controlling spoilage while preserving the nutritional value of the silage [11,14,21,22,33]. Inoculum helps to reduce the initial time in early stages of fermentation, when the forage may be otherwise colonized by undesired microorganisms. Some *Lactobacillus* spp. are used as inoculant cultures to initiate lactic acid production from glucose in silage without a delay, as it would otherwise start slowly because of the relatively low number of lactic acid-producing bacteria in herbage and the lag phase of bacterial growth kinetics. *Lactobacillus* species vary in their fermentation pathways. For example, *L. plantarum* is homofermentative and produces only lactic acid as the product. *L. buchneri* is heterofermentative and produces also acetic acid. Both organic acids acidify the solution to a pH below 5, which suppresses fungi and spoilage bacteria during anaerobic and aerobic phases. The *Lactobacillus* inoculant effect can also modulate the microbial community during ensiling [34]. Some *Lactobacillus* strains produce bacteriocins which suppress other bacteria, thus enhancing the lactic acid effect [35,36]. Guo et al. [37] tested over 100 strains of lactobacilli isolated from cow dung and rumen fluid as a supplemental inoculum for alfalfa ensiling. Only one isolate of *Lactobacillus plantarum* added at 1∙10^6^ viable counts/g silage, improved the silage digestibility and its abundance increased within the time course of 60 days (*L. plantarum* is now reclassified as *Lactiplantibacillus plantarum* [38]. The genus *Lactobacillus* has been revised by Zheng et al. [39] with the addition of 23 new genera, which improve the taxonomic distinction of lactic acid bacteria based on their phenotypic and genotypic traits and diversity). Three other isolates also had positive effects, recorded as sufficient lowering of the pH with time. The time course and small scale of silage batches (200 g fresh chopped alfalfa) make it challenging to extrapolate the results to large-scale ensiling. Successful *Lactobacillus* inoculation can dominate the fermentation and all other microorganisms in silage, possibly even reducing their diversity and richness [40].

The homofermentative *Lactobacillus acidophilus* has been successfully tested as an inoculum for leguminous silage [41]. *Lactobacillus* counts increased within two months in 400 g silage batches, and a combination of 1% malic acid and 1% citric acid with *L. acidophilus* lowered the pH and thus inhibited *Clostridium* and *Enterobacter* spp. and suppressed plant protein degradation. In general, the dominant lactic acid bacteria found in silage are those present in crops used for silage. The positive and negative effects of inoculants likely vary with the forage as well as the temperature and climate [34,42].

Other *Lactobacillus* species used successfully as starter cultures are *L. paracasei*, *L. hilgardii*, and *L*. *acidophilus*, sometimes in combination with *Pediococcus* spp. [25,43]. Multispecies inoculants of lactic acid bacteria have also been tested successfully [44]. Lai et al. [45] tested six commercial starter cultures, and they were shown to enhance the activities of lactic acid bacteria and suppress clostridia. All were mixed cultures and contained in varying proportions and abundance species primarily of the genera *Lactococcus*, *Pediococcus*, *Lactobacillus*, *Bacillus*, and *Leuconostoc*. Other benefits of these stimulant bacteria included the low pH range, which improved plant nutrient preservation. Analytical screening of silages with starter cultures yielded hundreds of metabolites [45,46], some with biological functions such as antioxidant and antimicrobial activities and biomolecules, which generally improved energy, nucleotide, and vitamin metabolism as compared to the control treatment without a starter culture.

Some studies with *Lactobacillus* starter cultures suggest differences in their effects on lactic acid production not only at the species but also at the strain level [47]. What traits make an efficient *Lactobacillus* starter culture is a complex issue without an unequivocal definition. Combination of starter cultures has usually given better ensiling results, but it also depends on the feedstock and the ensiling conditions, even the climate. Optimal starter cultures can be developed with selection techniques and molecular biology tools if the traits to be improved are defined. However, the extent of genetic modifications in silage starter cultures have limitations because silage treatment is an open bioprocess, it cannot be confined in a bioreactor, and the ingress of genetically modified bacteria or their DNA into animal and human nutrient cycles would be inevitable. Selection techniques to optimize bacterial traits may bypass the potential regulatory complexity.

Zhang et al. [48] reported slight enhancement of the biodegradation of insecticidal organophosphates in silage by some *Lactobacillus* starter cultures, but also other, mixed effects have been reported for lactobacilli [34,49,50]. In agriculture management practice, the multitude of combinations of pesticide formulations, crop plants, and geographical areas is considerable. The native silage microbiome represents the most important biodegradation capacity, but it may be enhanced with select starter cultures that are active in the acidic pH values [34,48]. Pesticides in silage are mostly residues of foliar applications of insecticides and fungicides. The residual concentrations vary because much of the residues of foliar pesticides wash off or dissipate with the weather and time. The residues may be present at low to mid microgram levels per kg dry matter [51]. Pesticide-degrading bacteria are common in agricultural soils and their transit to silage during harvest and ensiling is to be expected. Given the diversity of native microorganisms in silage, the capacity to degrade or mineralize pesticidal formulations cannot be specifically attributed to a specific bacterium or fungus that could discerned in the microbiome. Their degradative activities are suppressed by acid conditions, but slow degradation ensues in microenvironments especially if microbial metabolites have partially neutralized acidic conditions. Pesticide-degrading *Lactobacillus* spp. may exceptionally degrade pesticides in silage under acid conditions and may be good candidates for a mixed starter culture. *Lactobacillus*-mediated degradation of pesticide residues has been shown in laboratory-scale studies mimicking silo microcosms [34,48,49], but similar studies with full-scale silage would require innovative bioanalytical approaches to identify specific bacteria or fungi actively involved in pesticide transformations.

## 5. Potential Pathogens

Proper ensiling conditions are crucial not only for making high quality, palatable, and nutritive feed for livestock, but also for the biological safety of silage. Contaminated or poorly ensiled or stored silage (e.g., too slow of a drop in pH and/or aerobic conditions allowing undesirable bacteria to multiply) can be a source of pathogenic bacteria that may decrease animal performance, reduce the safety and quality of dairy products, and compromise animal and even human health [24,52]. Some pathogenic bacteria that can occasionally, or even frequently, be associated with silage are enterobacteria such as those in genera *Listeria*, *Clostridium*, *Bacillus*, and *Salmonella*. In some cases, the animal (and human) host may already have a compromised defense mechanism before clinical syndromes of infection by these pathogens.

*Listeria* spp. are naturally present in silage, decaying vegetable material, surface soil, and animal manure. They are regarded as opportunistic pathogens, especially harmful to people whose immunological condition is suppressed because of previous or existing health conditions. The route of transmission from silage is usually via farm animals or animal waste through to dairy products to humans. As reminded by Hishman [42], animal and human pathogens in ensilage are suppressed by lactic acid bacteria through acid fermentation and acidification during ensiling.

Clostridia are usually present in the starting material because their spores are ubiquitous in forage. The growth of clostridia in silage is undesired, because *Clostridium butyricum* and other saccharolytic clostridia form butyric acid upon lactate and glucose fermentation [22,24]. *Clostridium sporogenes* and other proteolytic clostridia decompose amino acids in protein catabolism involving deamination, decarboxylation, or oxidation-reduction (Stickland) reactions. The activity of clostridia is suppressed by the low pH conditions resulting from lactic acid fermentation [52,53]. This low pH effect is attributed to the toxicity of organic acids in undissociated form. In practice, pH 4 is sufficiently low to suppress clostridia and to ensure the long-term preservative effect if silage is not exposed to air. Some proteolytic enzymes from plant tissue and spore-formers including clostridia can be active below this threshold pH [54,55], but they are of minor significance in the overall silage preservation.

Silage contains opportunistic pathogens that can cause mastitis in dairy cows under poor hygiene and sanitation conditions; bacterial examples [56] include *E. coli* and select enteric pathogens in the genera *Pseudomonas*, *Klebsiella*, *Streptococcus*, *Staphylococcus*, and *Enterococcus*. Silage yeasts, such as in the genera *Candida*, *Trichosporon*, *Saccharomyces*, and *Rhodotorula*, can cause mycotic mastitis [57,58]. Unhygienic animal housing and barn environment during confinement season can be a persistent source of contagious pathogens from silage, spreading through feed residues, litter and bedding, manure, and fecal-oral contact. Possible contamination of silage by proteolytic bacteria due to poor sanitation and hygiene practices is an important management aspect of infection prevention. During silage removal and feeding, hands and utensils used in milking can become contaminated and transmit pathogens to the udder.

*Bacillus* spp. are common in soil and forage and are therefore sourced in silage. Because they are spore-formers they can survive acidity and passage through ruminal digestion system. *B. cereus* is among common food spoilage bacterium and causes gastroenteritis, which is a threat to livestock and human health. Its presence in milk usually indicates contamination, which can be controlled by strict hygienic milking and animal handling practices. The presence of *Salmonella* (salmonellosis) and other enteric animal and human bacteria and viruses in silage indicates manure or sewage sludge contamination associated with handling of harvest in the field or silage feed in the byre or cattle shed [59,60].

## 6. Bacteriophages

Bacteriophages, both temperate and virulent, have been found in bacteria in silage and they display considerable genomic diversity and host range that may impact carbon and nitrogen metabolism via phage-host interactions [61]. Some have been found in bacteria in the orders Campylobacterales and Enterobacterales. Phages specific to *Lactobacillus* [62] and *Listeria* [63] have been characterized. Some virulent phages in silage are known to have the *gldC* gene (glycine dehydrogenase) and may have other relevant genes linked to nitrogen cycling. The glycine dehydrogenase protein complex releases NH_3_ and CO_2_ from the amino acid, leading into synthesis of multiple other metabolites. Both temperate and virulent phages may contribute to the carbon cycle in silage by the *prsA* gene for the ribose-phosphate pyrophosphokinase [61]. The PrsA protein has multiple functions in mediating the pentose phosphate pathway and other critical metabolic steps. Virulent phages disrupt the host cell, releasing nutrients for recycling. Horizontal gene transfer among bacteria through transduction is also plausible in silage. Temperate phages may integrate into the bacterial genome and co-replicate in growth cycles. Phages may help the host survive via nutritional advantage or help the host defense against antibacterial compounds [64,65]. Much of the phage research on silage is still seeking to define functional aspects in the silage microbial community.

## 7. Fungi

Numerous filamentous fungi and yeasts have been identified in silage, including *Aspergillus*, *Fusarium*, *Geotrichum*, and *Penicillium* spp. and many others [28,66]. They live on plant materials as they secrete extracellular enzymes (e.g., lipases, cellulases, pectinases) that break down plant polymers. Fungi are mostly aerobic and associated with aerobic zones of silage. The presence of fungi in silage is unwelcome because of the possibility of mycotoxin production [67,68,69]. Examples of the most common mycotoxins posing a potential health risk are listed in Table 3. These are secondary metabolites in fungi, and their production is enhanced by prolonged wet weather conditions during harvest or poor storage of silage allowing exposure to air. Some act as mycoestrogens [69], analogous to phytoestrogens, which bind to estrogen receptors and may potentially have reproductive effects.

Aerobiosis can cause silage spoilage within a matter of days [69,70,71,72]. Aerobic silage layers provide for the bottom-growing yeasts (e.g., *Torulopsis* spp.) and the top-growing yeasts (e.g., *Hansenula*, *Candida* spp.). The genus *Saccharomyces* includes species in both categories. Yeasts in both categories can efficiently ferment and respire with sugars and both types can often be found in silage spoiled by aerobic conditions. Under aerobic conditions, yeasts can oxidize sugars, mineralizing them completely to CO_2_ and H_2_O. Duniere et al. [22] reported dominance of *Saccharomycetales* when grain silage was exposed to aerobic conditions. Yeasts compete against lactic acid bacteria for fermentable carbohydrates and can anaerobically produce ethanol (CH_3_CH_2_OH), which has no preservative value in silage [73]. Yeasts in aerobic silage layers cause spoilage because they can oxidize lactic acid, thus counteracting the role of lactic acid bacteria in sustaining acidification. Some yeasts can degrade pesticide residues in silage, but this activity has not been analyzed at depth because lactic acid bacteria in silage seem to be more important in this regard [48]. Duniere et al. [22] used next generation sequencing to follow microbiome changes in silage, first determined after 90 days of ensiling, which clearly demonstrated dominance of order II Lactobacillales especially. The succeeding aerobic exposure promoted proliferation of saprophytic fungi (order Hypocreales) and even some plant pathogenic fungi (*Sarocladium* spp.) were detected. Some yeasts are pectinolytic, as also are some Enterobacteriaceae. Their pectinase activity can lead to soft rot in silage, manifested as necrosis and cell wall decomposition of plant tissue.

## 8. Plant Fiber Polymers

Ensiling is intended to preserve all plant fibers until digested by livestock. Their relative proportions vary in different plant species. Cellulose (β-1→4-linked D-glucose units) is more readily degraded by cellulolytic bacteria and fungi than the other fibers in the silage, rumen, and monogastric digestion. Cellulose biodegradation also occurs under anaerobic conditions because the enzymatic reactions do not involve molecular O_2_ [74,75]. Cellulose is hydrolyzed stepwise by extracellular enzymes in silage microorganisms. Endo-acting cellulases hydrolyze linkages between sugar residues in the middle of the cellulose chain. Exo-acting cellulases cut linkages at the end of the β-1→4-linked D-glucose chains. The main products are glucose and cellobiose, which are further hydrolyzed into sugars and other oligosaccharides. The cellobiohydrolase (1,4-β-D-glucan cellobiohydrolase) enzymes produce sugars by hydrolytic cleavage of the non-reducing ends of the cellulose chains. Both bacteria and fungi (e.g., *Trichoderma* spp.) in silage have cellulolytic enzymes to break down structural cellulose. Cellulose degradation by bacteria and fungi, or additive cellulase enzyme, can release soluble carbohydrates, which may synergistically support activities of lactic acid bacteria in silage [76,77].

Pectin is a heterogeneous structural polysaccharide and is cross-linked to three-dimensional matrix of other plant cell wall polymers cellulose, hemicellulose, and lignin, which altogether make up the fiber content and help provide mechanical rigidity to the plant. The core structure of pectin is based on α-1,4-linked galacturonic acid subunits. Plant cell wall pectins are chemically complex, insoluble heteropolysaccharide polymers that require extracellular enzymes for initial decomposition. Several pectinases, differing in, for example, folding, ligand selectivity, and transporter mechanisms, have been characterized from soil bacteria and from phytopathogens especially [78]. Pectins are also major structural constituents in many fruits and berries. Pectins usually occur as partial methyl esters of α-1,4-linked D-polygalacturonate sequences that are interrupted with 1,2-L-rhamnose residues. The side chains usually consist of neutral sugars, such as L-arabinose, D-xylose, and D-galactose. The enzymatic breakdown of pectin involves multiple enzymes generically referred to as pectinases or pectinolytic enzymes. Pectinases include a range of pectin methylesterases, pectin lyases, transeliminases, and exo- and endopolygalacturonases that are involved in breaking down pectin into smaller entities. Multiple pectin-degrading bacteria are common in the microbial ensilage community [78,79], and their specific role and activity in the biodegradation is impossible to break down by species, intermediates, or metabolites.

Hemicellulose comprises β-1→4-xylan linkages, but the chemical constituents are heterogeneous, and the enzymatic degradation and depolymerization by hemicellulolytic microbes is slower than that for cellulose. Hemicellulose fibers are mixed polysaccharides, hydrolyzed mostly by extracellular enzymes in bacteria and fungi. The lignin fraction in the lignocellulose biomass is the most recalcitrant in the digestive system within the constraints of the transit time. Thus, lignin is indigestible, pectin is slowly digestible, and cellulose and hemicellulose are relatively readily hydrolyzed. The biologically available energy content of silage decreases with the total fiber content during digestion. Plant fiber polymers are linked in complex lignocellulose biomass, and their digestion initially commences slowly because of the lack of enzyme access to bound polymeric fiber molecules masked in the biomass.

## 9. Silage Moisture and Dry Matter

In general, the moisture content is 60–65% when the forage is ensiled, and this range supports lactic acid fermentation. High moisture content helps with compaction and exclusion of air that could otherwise result in spoilage. Initial moisture level of forage can vary between the extremes of flood and drought field harvest conditions. Excess moisture (>70%) leads to seeping and loss of nutrients from silage and can sometimes be associated with clostridial fermentation. Liu et al. [80] demonstrated major differences in metabolites produced in alfalfa silage at 60% and 80% moisture content. Volatile organic compounds were more dominant at the higher moisture content; these included terpenes, inorganic and organic sulfides, and nitrogen oxides. Organic sulfides were especially indicative of protein breakdown due to anaerobic biodegradation of S-compounds such as methionine, cysteine, homocysteine, taurine, and glutathione. The presence of N-oxides (nitric oxide and nitrous oxide) indicates denitrification activity or anaerobic protein decay with subsequent aerobic exposure whereby NH_3_ is oxidized to NO_2_^−^ and NO_3_^−^. The moisture content at 80% provided poor conditions for lactic acid fermentation [80]. *Lactobacillus* and *Pediococcus* spp. were much more abundant at 60% moisture content and silage quality was greatly improved.

Dry matter (i.e., plant biomass) is the solid phase of the energy and carbon source for animal feed and growth and usually controlled by moisture adjustment, if possible. Therefore, almost any biological activity that consumes or modifies silage impacts dry matter, such as the structural fiber biodegradation [81]. Silage moisture is, of course, connected to silage dry matter: categorically, >70% moisture content equals < 30% dry matter. The dry matter content varies with feedstock crop within ±10%. Silage with low dry matter content leads to nutrient and carbon losses in seepage; conversely, high dry matter content is difficult to consolidate into anaerobic layers. Dry matter content decreases upon ensiling because of fermentation and other microbial metabolism. On a weight basis, 15–20% dry matter losses have been reported in ensilage over three months [34], attributed to soluble products of microbial metabolism. Heterofermentation and mixed acid fermentation pathways result in release CO_2_, causing more loss of dry matter as compared to homofermentation. Formation of CO_2_ represents a complete loss of carbon from silage.

## 10. Chemical Additives

Several chemical additives are available to control the process and quality of silage (Table 3). The acidification of harvested forage to pH 3.6 with the use of dilute mineral acids (HCl or H_2_SO_4_), known as the AIV process, used to be a common practice, especially in the Scandinavian countries, starting in the 1930s [82,83,84]. This AIV process (AIV stands for the 1945 Nobel Laureate (Chemistry) A.I. Virtanen, credited with the development of the process) was based on the premise that pH 3.6 is sufficiently low to preserve the material and inhibit microbial spoilage. In the AIV ensiling, mineral acids (HCl and H_2_SO_4_) were initially used but were subsequently replaced with a formula of formic acid, ammonium formate, and water.

Carbohydrate-rich additives have been added to enhance the fermentation; these materials include sugars, molasses, cereals, beet or citrus pulp, whey, and potatoes. This kind of additive effect is often a question of the cost of material and transportation weighed against the beneficial, sometimes perceived, effects of improved lactic acid fermentation. Nitrogenous supplements in the form of urea can be beneficial in ensiling low-N forage such as maize. In contrast, fermentation inhibitors such as mineral acids in the AIV process have been used to inhibit plant and microbial enzymatic activity [32]. In practice, biological activity is greatly suppressed due to acid conditions but not completely inhibited. The low pH in the silage process effectively eliminates proteolytic decomposition of plant biomass. Mineral acid mixtures have been tested and marketed in several formulations under different trade names, mainly as mixtures of hydrochloric and sulfuric acid or hydrochloric and phosphoric acid [24]. Trademark protection is current with supplemental liquid formulations available commercially. Different commercial formulations of inoculants are optimized for specific crop plants, such as alfalfa, corn or grass, or organic farming. As a counterweight, ensiling is an open bioprocess with little to no direct control over microbial activity in silo other than the low pH. Much of the practice in ensiling has come from empirical and agriculture industry-sponsored studies, and feedback from farmers over the decades.

Formic acid (HCOOH) has also been used as a silage additive to neutralize the buffering effect of herbage and lower the pH to below 5, which effectively eliminates many of the spoilage microorganisms in silage. Undissociated formic acid also inhibits spoilage microorganisms, thereby restricting the profile of organic acid fermentation and conserving carbohydrate components of silage. Acetic and propionic acids have also been evaluated but have not proven as useful as formic acid. By contrast, mineral acids were primarily used for acidification and have little direct effect on spoilage microorganisms.

Formaldehyde is also added because the compound is bacteriostatic and can protect plant protein from microbial decomposition in the rumen. The additive must be carefully dosed because excess formaldehyde (as free formaldehyde, HCHO, structurally H_2_C=O) would be deleterious to silage fermentation and rumen microorganisms. Formaldehyde links readily with terminal amino, hydroxyl, amide, guanidyl, sulfhydryl, indole, phenyl, and imidazole groups in amino acids by forming a methomyl derivative, which undergoes condensation reactions with time to produce stable methylene cross linkages between peptides. Other silage additives acting as inhibitors and tested with varying degrees of success are, for example, sulfur dioxide, sodium metabisulfite, ammonium metabisulfite, and Na-chloride. Empirically, bisulfite inhibits protein breakdown and the loss of nitrogen as NO_2_ and suppresses the formation of acetic acid and butyric acid. Bisulfite-treated silage has more nitrate and carotene. Na-chloride addition has been used in the top layers to inhibit aerobic microbial activity and reduce aerobic spoilage and mycotoxin levels [84]. Antimycotic effects have also been noted for propionic acid [85]. Compounded additives rather than a single additive may be more effective for the desired effect in silage treatment [86]. As with all combinations of additives and forage types, the cost–benefit analysis must be considered before implementation in large-scale silage systems.

Additions of antibiotics to silage were of interest decades ago, when it was discovered that some had differential effects and increased the fermentation in selected cases [87]. Antibiotic-containing feed has a long history of practice because these compounds are known to enhance animal growth and milk production. Contrastingly, the public is critical of this practice because it may spread antibiotic resistant microbes in the environment, often in agricultural fields. Antimicrobial resistance genes have been detected in silage [88,89,90]. These findings suggest a route of emergence of antimicrobial resistance in food-producing livestock leading to human food chains [91]. They can lead to wide occurrence of antimicrobial resistance in agricultural settings [92,93,94]. The problem has since been recognized as a major public health issue and use of these antimicrobial additives is regulated in many countries. An EU-wide ban on the use of antibiotics as growth promoters (Feed Additives Regulation 1831/2003/E) in animal feed came into effect on 1 January 2006. From that point forward antibiotics have only been allowed to be added to animal feed for veterinary purposes. In the U.S., the Veterinary Feed Directive (VFD) final rule published in February 2024 requires veterinarians to issue all VFDs within the content of a veterinarian-client-patient-relationship (VCPR) and specifies the key elements that define the VCPR.

## 11. Ruminal Effects

It is rare to find detectable concentrations of methane in silage because the low pH suppresses methanogens. Protozoa are not found in silage but are stable constituents in the rumen. Some silage diets may suppress methane formation in the rumen [95,96,97,98], indicating major changes in the rumen microbiome and metabolites. Thus, the type of forage, ensiling process, and inoculants may impact ruminal methanogenesis [99]. Acetate, usually the second most abundant short-chain fatty acid in silage, has a key role in methanogenesis. Acetoclastic methane formation converts the methyl group in acetate to CH_4_ and the carboxyl group (-COOH) is released as CO_2_. There also are other methylated precursors to methane formation, but their concentrations are insignificant compared with acetate and carbon dioxide in silage and rumen. The syntrophic pathway proceeds via acetate oxidation to CO_2_, which is reduced to CH_4_ with H_2_ as the reductant in the hydrogenotrophic methane formation. Silage rich in starch can favor amylolytic bacteria, shifting volatile fatty acid formation from acetate to propionate and even butyrate [100]. Starch fermentation is faster than plant fiber digestion. The shift in fermentation decreases the pH and reduces hydrogen availability to rumen methanogens, because these archaea are also suppressed by the low pH. The microbial interactions, including cross-feeding and interspecies H transfer in the rumen, are complex and interwoven. Methane suppression effects are difficult to modulate in vivo and may have adverse consequences in the health of the animal as, for example, bouts of ruminal acidosis or effects on feed intake or lactation.

With microbial fermentations, the ensiling process also yields volatile compounds to a varying degree [101]. Losses of carbon in volatile compounds also involve changes in the taste of the silage and a decreasing dry matter content [72]. Lactic acid is odorless, but some lesser acids have a distinct smell: vinegar odor due to acetic acid, which is usually the second most common organic acid in silage; and a strong, unpleasant odor caused by butyric acid produced in clostridial fermentation [72,102]. Acetic acid contributes to the lowering of the pH, suppresses yeasts, and helps stabilize silage when exposed to air [103]. In general, volatile compounds affect the quality, and acceptability and olfactory responses of animals to the feed, and rejection in the worst-case scenario [104]. Thus, inoculation of lactic acid bacteria can be used to modulate biochemical pathways of plant sap fermentation in the ensiling process to help ensure that lactic acid is the dominant metabolite [24,33].

Several chemical additives so suppress methane formation in ruminants have been discussed and tested over the years [105]. Examples of additives in cattle feed include (i) volatile oils extracted from plant biomass, mostly terpenes and phenylpropanoids; (ii) nitrate as an alternative sink to H but this may result in nitrite accumulation and animal intoxication and potential risk to human health; (iii) halogenated C-compounds (mostly C1) such as dichloromethane, chloroform, trichloroacetamide, trichloroethyl adipate, and carbon tetrachloride. They can effectively block the function of corrinoid enzymes and the methyl group transfer in methane formation, but their use is banned worldwide because of their animal and human toxicity; (iv) nitrooxycompounds, most commonly 3-nitrooxypropanol (3-NOP), which inhibit specifically the methyl-CoM reductase activity in methanogens [99,106]. Whether the inhibitors are added in the silage or any other feed varies with the livestock operation. While the concentration or dose of the inhibitor is relatively small, its addition to the silage is at risk because of the active microbial biotransformation of the inhibitor molecule may modify or degrade the additive partially or completely, resulting in the lack of its biological target activity. Direct feeding is the choice for additives that are biologically not stable in silage.

## 12. Silage Stability

Silage stability or bunk life refers to the resistance to aerobic spoilage upon exposure to air. This situation of aerobic spoilage occurs in the silo face or in the feed bunk. Aerobic conditions promote yeasts and fungi, but their activity is sensitive to the combination of high lactic acid content and low pH conditions. High ammonia level is also prohibitive to eukaryotic microorganisms, and this effect is sometimes used to increase the bunk life through addition of ammonia to silage, a process called ammoniation [107,108,109]. Aerobic spoilage is a combination of aerobic microbial consumption of fermentation end products and plant sugars and other carbohydrates to heat, CO_2_, and H_2_O. Consequently, nutrients and dry matter are lost from the silage. The bulk of acid balance can be lost due to aerobic respiration, resulting in an increase in the pH to circumneutral values. Temperatures in aerobic zones of silage may rise in excess of 35 °C to 45 °C due to aerobic respiration, resulting in losses of dry matter and acid balance [110]. Spoilage microorganisms may be toxic because of their pathogenicity, or they may make the silage more conducive to pathogenic microbes.

Increased bunk life minimizes the presence of mycotoxins in silage, which can otherwise be a considerable risk to animals receiving silage in their feed [111,112]. Table 4 lists examples of mycotoxins that have been found in spoiled silage and are hazardous to animal and human health. In addition to mycotoxicosis, animal and human bacterial pathogens can proliferate (e.g., *Listeria* spp.) under aerobic conditions. Bunk life is also subject to aerobic microbial respiration on silage, which causes accumulation of crusty or slimy layers that are usually inedible by animals. It has been speculated that zoonotic pathogens such as Shiga toxin-producing *E. coli* (STEC) can be spread via silage feeding [113], but evidence to date is questionable for this modus of distribution. It is likely that disease-causing bacteria such as STEC are suppressed in silage due to the production of organic acids and low pH during ensiling.

Global climate change is predicted to affect forage crops and soil microbial metabolism, and these effects likely impact forage yields, silage fermentation, bunk life, metabolites, and eventually spoilage [114,115]. As an example, Guan et al. [111] showed in a bench-scale study that an increase in the ensiling temperature from 30 °C to 45 °C changed the dynamics of homofermentation to the less efficient heterofermentation. However, the effects of slow, subtle temperature changes on the silage quality and microbiome are experimentally very difficult to substantiate.

## 13. Human Health Risk

The filling of the upright silo exposes farm workers to potential health hazards because the fermentative decomposition of plant materials produces CO_2_, NH_3_, and several toxic gaseous oxides of nitrogen [116]. Nitric dioxide (NO_2_) is the most prominent of the N-oxides. Concentrations > 2000 ppm NO_2_ have been detected in some silos [117]. NO_2_ is heavier than air and hence it accumulates on the surface of silage in closed space and on the floor level of feed sheds or barns. Exposure to NO_2_ causes silo filler’s disease, which is caused by displacement of O_2_ with NO_2_ in the breathing zone [118,119,120,121]. Similarly, fresh silage produces carbon dioxide which may accumulate to toxic elevated levels in closed space. Anaerobic microbial metabolism at excessive moisture content (>70%) can result in the formation of toxic volatile sulfides (e.g., hydrogen sulfide H_2_S, dimethyl sulfide CH_3_SCH_3_, methyl mercaptan CH_3_SH). Fatal occupational risks have also been reported for being trapped under collapse of high silage piles when extracting feed (https://www.pubs.ext.vt.edu/DASC/DASC-103/DASC-103.html (accessed on 21 September 2025).

In addition to chemical risks, toxin genes of bacterial pathogens have been found in silage, including those of *Clostridioides difficile* and *Clostridium botulinum*, and they have also been detected in manure [52,122]. Potential zoonotic pathogens and spore-formers especially (e.g., *Clostridium*, *Clostridoides*, *Bacillus* spp.) in silage can survive prolonged storage and transit to manure. Biosecurity, sanitation, and hygienic practices with handling and feeding silage to livestock and manure management are key in avoiding accidental transmission of zoonotic and spore-forming pathogens to dairy and other animal products.

## 14. Concluding Remarks

Silage is fed to livestock as a feed supplement and as an alternative to dry hay during the winter season and extended drought. High-intensity dairy and beef farms and small-scale family operations depend on silage year-round for livestock feeding. Dairy and beef cattle have different nutritional requirements for silage and digestibility. Silage can also be used as a feed supplement to sheep, goats, and horses when outside pastures are not accessible or out of season. For the former, the chop length must be shorter, thus requiring modified ensiling process. Silage has primarily been developed for cattle, and less information is available on optimal silage conditions for other livestock ruminants and non-ruminants.

Much of the current knowledge on ensiling has been achieved from empirical trials and research. The silage industry has commercialized chemicals, inoculants, other additives, technology, and machinery for ensiling, and has kept pace with advancements over the years. Ensiling captures the nutrient and energy content of forage and herbage that would otherwise be unavailable for farm animals due to physical or climatic barriers. The uninoculated microbiome of silage reflects the microbial community of plant material used as a feedstock. Ideally silage is acidic at pH 4–5 and has bacterial activity that is responsible for silage acidification. Much of the nutritional value, especially the N-content, is preserved in silage. Lactic acid fermentation is key in ensuring silage preservation and palatability and several biological and chemical additives are available to control the time course and silage quality.

Intercropping feedstock (e.g., maize + legume) may improve the nutritive content of silage. Important parameters include the protein (or amino acid) content and nutritive value as well as fiber and dry matter content. Research into these biologically interactive, multifaceted components may yield improvements in the silage quality and quantity. Optimizing the combination of intercropping and starter culture selection can enhance animal growth, possibly even bring about savings in minor and trace element and cofactor supplements and crop land area.

The relationship between silage quality and composition and ruminal metabolism needs further analysis. Both carbon dioxide and methane formation in the rumen (paunch) represent loss of carbon, which could otherwise be used toward the animal’s energy and biomass growth. By a factor of about 28 on a weight basis, methane is more potent than carbon dioxide as a greenhouse gas but has a shorter half-life in the atmosphere of about 12 years, *vs.* centuries to millennia for CO_2_.

Chemical additives (e.g., 3-NOP) in the feed to suppress methanogens in the paunch have been met with some skepticism because the residues and their potential effects on animal and human health aspects are not fully understood. Their effects on the total digestive system—the rumen, reticulum, omasum, and abomasum—have yet to be fully explored. The suppression of methane formation also ties in with climate change, which in the course of time affects plant growth, diversity, and nutritive composition. These subtle changes in plant feedstock affect silage composition, but the potential effects on silage microbial metabolism and feed quality and palatability are unknown.

## Figures and Tables

**Table 1:** Different phases of ensiling. Information retrieved and modified from references cited in this paper. Some references claim that silage may be preserved for the feed-out for up to 3 years if perfectly packed and stored.

Post-Ensiling Phases	Description
Pre-seal phase, 1 to 2 days	Aerobic respiration by plant cells and microbes depletes the remaining oxygen and carbon dioxide and H_2_O are formed, and heat evolution is noticeable. The length depends on the packing and compaction of the forage. A prolonged aerobic phase can result in excessive heat, loss of dry matter, invasive yeasts and filamentous fungi, and mycotoxin formation. The initial pH starts to decrease.
Lag phase, 2–5 days	Plant and microbial enzymes break down complex carbohydrates in plant cells into sugars that enter fermentative pathways. Partial degradation of plant proteins. The pH decreases to 5.7–5.5.
Active fermentation phase, up to 2 weeks	Oxygen is depleted and fermentation takes place under anaerobic conditions. The pH is decreased to <pH 5.7, which is favorable for lactic acid bacteria. Lactic acid is produced along with lesser amount of acetic acid and minor amounts mannitol and ethanol. Lactic acid accounts for 70% or more of the total acids in silage. Silage temperature is slightly elevated and the initial moisture content decreases with bacterial activity. The active fermentation phase lasts for up to two weeks.
Stable phase, after about 3 weeks	Stable phase after ensiling. Silage pH is in the range of 3.8 to 4.2, silage is stabilized and preserved, and bacterial activity is declined because of the lack of soluble available substrates. Changes in silage continue throughout the storage, usually due to continued bacterial action and plant enzymes breaking down cellular protein. The temperature is stable at about 20 °C.

**Table 2:** The main end products of sugar fermentation. The products of mixed acid fermentation vary with the substrate and specific microorganism.

Pathway of Lactic Acid Fermentation	Substrate	Products
Homofermentation	Glucose or fructose	2 × lactic acid
	Pentose	Lactic acid + acetic acid
Heterofermentation	Glucose	Lactic acid + ethanol + CO_2_
	3 × Fructose	Lactic acid + 2 × mannitol + acetic acid + CO_2_
	Pentose	Lactic acid + acetic acid
Mixed acid fermentation	Glucose	Lactic acid, acetic acid, ethanol, formic acid, succinic acid, CO_2_

**Table 3:** Examples of mycotoxins found in silage, potentially causing toxicosis in animals. Silage fungi in other genera may also produce some of these mycotoxins.

Mycotoxin Group	Genus	Manifestations in Animals
Deoxynivalenols	*Fusarium*	Food refusal, diarrhea, reproductive failure
Aflatoxins	*Aspergillus*	Carcinogenic effects
Zearalenones	*Fusarium*	Estrogenic effects
Fumonisins	*Fusarium*	Feed refusal, liver disease
T-2 Toxin (trichothecenes)	*Fusarium*	Gastroenteritis and intestinal hemorrhage
Tremorgens	*Aspergillus*	Anorexia, diarrhea
Ochratoxins	*Penicillium*	Diarrhea, kidney damage
Patulins	*Fusarium*	Hemorrhagic disease

**Table 4:** Examples of silage additives.

Class	Additive or Ingredient	Mode of Action	Examples
Acidifiers	Mineral acids	Lower the pH and prevent proteolytic decomposition (ammonification)	Hydrochloric–phosphoric acid, hydrochloric–sulfuric acid
Acidifiers	Organic acids	Lower the pH and eliminate spoilage or competing microorganisms	Formic acid
Fermentation inhibitors	Sterilants	General inhibition of the microbial population; protein stabilization	Formaldehyde
Fermentation stimulants	Substrates, enzymes, inoculants	Promote carbohydrate fermentation; release fermentative substrates from non-fermentative sources; promote lactic acid bacteria	Molasses, cellulolytic enzymes, lactobacilli
Antimicrobials	Antibiotics and other antimicrobial agents	Inhibit spoilage microorganisms (e.g., clostridia)	Bacitracin (controversial), Na-chloride, NaNO_2_
Nutrients	Energy substrate; N-compounds	Improve nutritional value of silage	Cereal, urea

## Data Availability

No new data were created or analyzed in this study. Data sharing is not applicable to this article.
